# Derivation of a prototype asthma attack risk scale centred on blood eosinophils and exhaled nitric oxide

**DOI:** 10.1136/thoraxjnl-2021-217325

**Published:** 2021-08-06

**Authors:** Simon Couillard, Annette Laugerud, Maisha Jabeen, Sanjay Ramakrishnan, James Melhorn, Timothy Hinks, Ian Pavord

**Affiliations:** 1 Respiratory Medicine Unit, Nuffield Department of Medicine, University of Oxford, Oxford, UK; 2 Faculté de médecine et des sciences de la santé, Université de Sherbrooke, Sherbrooke, Quebec, Canada; 3 Division of Immunology, Sanofi Genzyme Norway, Oslo, Norway; 4 School of Medical and Health Sciences, Edith Cowan University, Joondalup, Western Australia, Australia

**Keywords:** asthma, exhaled airway markers, eosinophil biology, asthma epidemiology, respiratory measurement, pulmonary eosinophilia, clinical epidemiology, allergic lung disease

## Abstract

Reduction of the risk of asthma attacks is a major goal of current asthma management. We propose to derive a risk scale predicting asthma attacks based on the blood eosinophil count and exhaled nitric oxide (FeNO). Biomarker-stratified trial-level attack rates were extracted and pooled from the control arms of the Novel START, CAPTAIN, QUEST, Benralizumab Phase 2b, PATHWAY, STRATOS 1–2 and DREAM trials (n=3051). These were used to derive rate ratios and the predicted asthma attack rate for different patient groups. The resultant prototype risk scale shows potential to predict asthma attacks, which may be prevented by anti-inflammatory treatment.

## Introduction

Assessment and reduction of the risk of attacks are a major goal of asthma management.^1^ However, our ability to do this is limited for several reasons. First, the extent to which the risk associated with clinical characteristics is independent of the inflammatory phenotype has not been defined. Second, some acknowledged risk factors are difficult to identify and/or modify, for example, non-adherence and obesity, respectively. Third, some parameters can be modified independent of an effect on asthma attacks; for example, symptom burden improves following bronchodilator monotherapy without an effect on asthma attacks.^2^ These limitations mean that a precise estimation of the risk of asthma attacks and the likely benefit of treatment is not possible.

Recently, five analyses of clinical trials across the spectrum of asthma severity have assessed the independent relationship between blood eosinophils, fractional exhaled nitric oxide (FeNO) and the risk of asthma attacks.^3–7^ Collectively, these studies show that the prognostic importance of these biomarkers is similar in strength and additive to the independent risk seen with more established risk factors such as a history of an attack in the last year and Global Initiative for Asthma (GINA) treatment step.^8^ In four out of the five studies, the prognostic value of blood eosinophils and FeNO was additive.^3 5–7^

These findings suggest that the blood eosinophil count and FeNO could form the basis of a useful risk scale analogous to those that have had a large impact in cardiovascular medicine.^9^ We have explored this hypothesis by developing a prototype risk scale.

## Methods

We designed a scale presenting the modifiable risk of asthma attacks associated with blood eosinophils and FeNO on the background of the unmodifiable risk associated with GINA treatment step, a recent history of an asthma attack and the presence of less modifiable risk factors. Asthma attacks were defined as episodes of acute asthma requiring treatment with systemic steroids ≥3 days and/or hospitalisation.

We used control arm data^3–7^ from the trials described in the supplementary table (see online supplemental file 1) to derive frequency-weighted rate ratios of asthma attacks by biomarker combinations using established cut points for blood eosinophil counts and FeNO (table 1). Individual trial rate ratios were calculated as follows: [(absolute asthma attack rate for subgroup 1)×(frequency *n_1_*)] ÷ [(frequency-weighted mean for the remaining subgroups 2–9)×(Σ(*n_2 to 9_*))]. Aggregate rate ratios (rightmost column of table) were calculated as frequency-weighted means of the individual trial’s rate ratios for each biomarker combination. In effect, an aggregate rate ratio is a mean fold change in the asthma attack rate for patients with that biomarker combination compared with others.

10.1136/thoraxjnl-2021-217325.supp1 Supplementary data

**Table 1:** Biomarker-stratified data and rate ratios derived from included trials

Blood Eos (×10^9^/L)	FeNO (ppb)	Novel START^4^			CAPTAIN^5^			Pooled AZ trials: Benralizumab 2b, PATHWAY, STRATOS 1–2^7^			QUEST^6^			DREAM^3^			Aggregate data for the prototype risk scale	
		Step 1 asthma; low risk; 9% with attack in past 12 months			Step 4 asthma; high risk; 62% with attack in past 12 months			1% step 3 asthma, 50% step 4 asthma, 49% step 5 asthma; high risk; with attack in past 12 months			47% step 4 asthma, 53% step 5 asthma; high risk; with attack in past 12 months			Step 5 asthma; high risk; with attack in past 12 months				
		N†	Attack rate‡	Rate ratio	N	Attack rate‡	Rate ratio	N†	Attack rate	Rate ratio	N	Attack rate	Rate ratio	N	Attack rate	Rate ratio	N	Rate ratio
<0.15	<25	18	0.05	0.98	228	0.85	0.54	199	0.58	0.81	106	0.56	0.52	23	1.98	0.76	574	**0.65**
	25–<50	23	0.00	0.00	40	0.10	1.11	82	0.46	0.64	35	0.62	0.61	(9)	(1.78)	(0.71)	180	**0.66**
	≥50	8	0.00	0.00	17	0.15	1.74	23	0.57	0.81	21	0.53	0.53				69	**0.86**
0.15–<0.30	<25	19	0.07	1.50	240	0.07	0.82	191	0.56	0.76	96	0.82	0.80	12	1.54	0.59	558	**0.81**
	25–<50	42	0.02	0.36	87	0.07	0.79	173	0.67	0.96	53	1.14	1.17	(23)	(2.70)	(1.07)	355	**0.88**
	≥50	32	0.01	0.24	24	0.12	1.43	52	1.29	1.93	25	0.48	0.47				133	**1.16**
≥0.30	<25	4	0.30	6.35	248	0.11	1.29	102	0.58	0.82	89	0.84	0.84	18	1.95	0.75	461	**1.12**
	25–<50	22	0.00	0.00	147	0.09	1.00	133	0.87	1.30	97	1.24	1.31	(66)	(3.08)	(1.22)	399	**1.12**
	≥50	51	0.13	4.40	66	0.18	2.14	107	1.01	1.53	98	1.78	2.12				322	**2.29**
Analysed		219	0.05	1.00	1097	0.09	1.00	1062	0.70	1.00	620	0.99	1.00	151	2.52	1.00	3051	1.00
Missing*		4			121			120			14			4			262	
Total		223			1218			1182			634			155			3313	

Aggregate ratios in bold (rightmost column) are those included to derive the prototype risk scale: in effect, an aggregate rate ratio is a mean fold change in the asthma attack rate for patients with that biomarker combination. Numbers between brackets were extracted to calculate frequency-weighted rate ratios but were not used to derive the scale, as this analysis was stratified using only two cut points for fractional exhaled nitric oxide (FeNO <25 or ≥25 ppb).

Blood Eos, peripheral blood eosinophil count; *n*, number of patients.

*Missing data were excluded from analyses.

†For Novel START and the pooled AstraZeneca (AZ) trials, we regrouped the data of patients with a baseline FeNO of 20–<50 ppb into our 25–<50 ppb group, as the difference of 5 ppb in FeNO is not clinically relevant.

‡For both the Novel START and CAPTAIN, only the percentage of patients with one or more severe attack(s) in the 52 weeks of follow-up was reported, so we imputed the annualised rate as ^–^log_10_(1 − %incidence).

We used asthma attack rates from a US population study involving 222 817 patients to derive a predicted asthma attack rate by GINA step.^8^ We further stratified by a history of an asthma attack in the last year (which we assumed increased risk by a factor of 2.8)^8^ and the presence of two or more additional potential risk factors (which we assumed increased risk by a factor of 1.3). Our estimate of the additional risk associated with two or more additional potential risk factors was based on the difference in asthma attack rates in the CAPTAIN population,^5^ who had persistent symptoms and airflow obstruction, compared with the Novel START population,^4^ who had neither.

To populate each cell of the prototype risk scale, the reference rate for GINA treatment steps 1, 2, 3, 4 and 5 was multiplied by the appropriate risk pertaining to that group for example, the figure’s rightmost column’s rates are calculated as [aggregate biomarker-stratified rate ratio] × [GINA treatment step-specific attack rate] × 2.8 × 1.3.

A frequency-weighted intraclass correlation coefficient (two-way mixed model for absolute agreement of single measures) and 95% CIs were computed between the predicted and observed asthma attack rates using the derivation trials in SPSS V.27.

## Results

**Figure 1:**
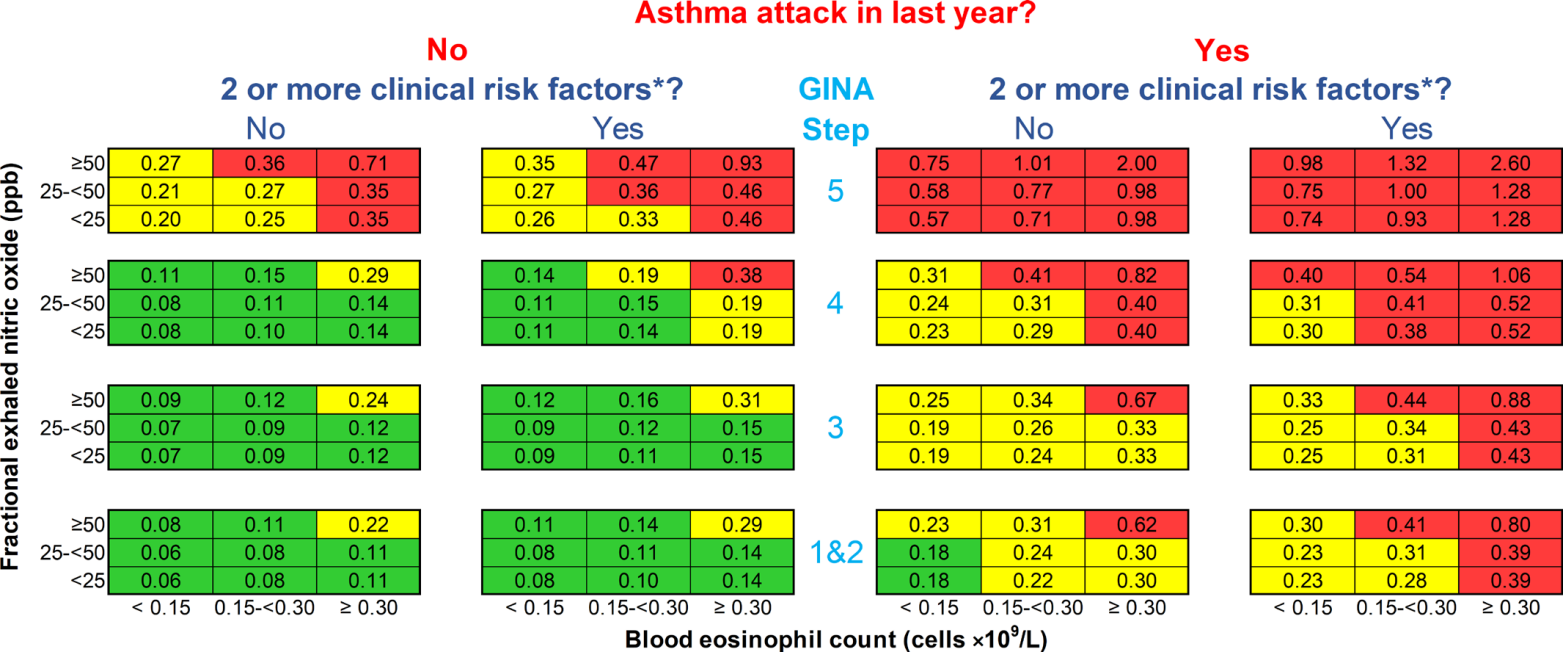
Prototype asthma attack risk scale. Numbers in each cell are predicted annual asthma attack rates for patients over the age of 12 if treatment is not changed. An asthma attack is an episode of acute asthma requiring treatment with systemic steroids ≥3 days and/or hospitalisation. The blood eosinophil count is contemporaneous or the highest result in the last 12 months; fractional exhaled nitric oxide level is contemporaneous. *Risk factors are defined by the Global Initiative for Asthma (GINA) guidelines^1^: poor symptom control (Asthma Control Questionnaire score ≥1.5), low lung function (forced expiratory volume in 1 second <80% predicted), adherence issues, reliever overuse (>200 dose of salbutamol cannister/month), intubation or intensive care unit admission for asthma previously, comorbidities (one of chronic rhinosinusitis, obesity and psychiatric disease) and environmental exposures (one of smoking, allergen and pollution).

The resulting prototype risk scale is shown in the figure 1: each cell represents the predicted annual asthma attack rate for a given scenario if treatment is not changed. The predicted asthma attack rates range from 0.06 to 2.60 per year; they are comparable to observed attack rates in the derivation trial control patients (intraclass correlation coefficient: 0.83 (95% CI 0.78 to 0.86)).

## Discussion

We designed a prototype risk scale based on trial-level data that shows potential to predict asthma attacks which may be modified by anti-inflammatory treatment. As is the case with cardiovascular risk, the relative risk associated with biomarkers was consistent across populations, but the absolute risk conferred by type 2 airway inflammation was greater in a population at higher background risk.

The fact that blood eosinophils and FeNO provide additive prognostic information is predictable, as both biomarkers provide different and complementary mechanistic information: FeNO reflects airway type 2 activity and the chemotactic pull to the airways, while blood eosinophils reflect the systemic pool of available effector cells and circulating interleukin 5.^10^ In contrast, symptom scores do not correlate with airway inflammation nor with airflow limitation^10^ and do not reliably predict exacerbations when the inflammatory phenotype is considered.^11^

An important feature of the prototype risk scale is that it centres attention on biomarkers that are not only closely associated with the mechanism of asthma attacks but are also easily modified with therapy directed against this mechanism. For example, the excess risk of asthma attacks associated with the highest biomarker combination compared with the lowest was effectively removed by low-dose inhaled corticosteroids (ICS) in mild asthma,^4^ an increased dosage of ICS in moderate asthma^5^ and biologics in severe asthma.^3^ In many cases, this reduction in risk is associated with a proportionate reduction in biomarkers.

We emphasise that the proposed risk scale is a prototype and several assumptions have been made in its derivation. First, there were some inconsistencies in the relationship between FeNO and the risk of asthma attacks in the mild asthma population,^4^ which likely reflect the small sample sizes. However, a difference in the mechanism of asthma attacks or a relatively greater prognostic value of FeNO in ICS-treated patients cannot be excluded. Larger studies are required to investigate these possibilities. Second, we categorised risk factors, and since the independent risk conferred by these risk factors over and above that associated with type 2 biomarkers is unknown, we derived the multiplier for having ≥2 risk factors by comparing the Novel START^4^ and CAPTAIN^5^ populations. The resultant multiplier of 1.3 suggests that the independent impact of these factors is modest, but further work is needed to confirm this. Third, although the biomarker-stratified rate ratios were adjusted for each other, we concede that the other covariates were not perfectly adjusted for one another. Fourth, the prototype features categories rather than the absolute values of blood eosinophils, FeNO and clinical risk factors. We did this as this was the only data available to us. It also allowed us to tabulate risk across the spectrum of patients and biomarkers in an accessible way. This approach has been very successful in cardiovascular risk reduction, but we acknowledge that there may be better ways of representing the continuous risk associated with these factors.

We speculate that a risk scale based on this prototype could facilitate better treatment decisions by doctors and patients by providing a framework for a preventive, treatable, trait-based management. This hypothesis needs to be tested, and it is also important that the scale is refined using individual patient data from large and well-characterised populations.
