# Supplementary material for: Derivation of a prototype asthma attack risk scale centred on blood eosinophils and exhaled nitric oxide
Source: Thorax. 2021 Aug 6;77(2):199–202. doi: 10.1136/thoraxjnl-2021-217325 (PMC8762000; doi:10.1136/thoraxjnl-2021-217325)
Supplement: Supplementary data [file thoraxjnl-2021-217325supp001.pdf]

Derivation of a prototype asthma attack risk scale centred on blood eosinophils and exhaled nitric oxide

Simon Couillard, Annette Laugerud, Maisha Jabeen, Sanjay Ramakrishnan, James Melhorn, Timothy SC Hinks, Ian D Pavord

Online data supplement

Table of Contents

Content	Page
**Supplementary Table.** Biomarker-stratified randomised controlled trials analysed to derive the prototype risk scale	E3
**Supplementary References**	E6

SUPPLEMENTARY TABLE
Biomarker-stratified randomised controlled trials analysed to derive the prototype risk scale

Trial name (registration number)[Ref#] Study design	Arm included (*n* / total *N*)	Key Inclusion criteria	GINA Step: No (%)	ACQ mean score	FEV1 % predicted	PostBD change in FEV1 (%)	Blood Eos ($x10^9/L$)	FeNO (ppb)
Novel START (ACTRN12615000999538)[1] 52-week, randomised, open-label, parallel-group, controlled trial	Salbutamol as needed (223/668)	SABA monotherapy in previous 3 months; SABA use on at least 2 occasions and an average of ≤2 occasions per day in the previous 4 weeks; no minimum requirement for SABA use in those with severe exacerbation in last 12 months	Step 1: 219 (100)	1.1 (0.7)	89 (14)	nd	0.3 (0.2)	40 (5-235)*
CAPTAIN (NCT02924688)[2] 52-week, phase IIIA, randomised, double-blind, active-controlled, parallel-group double versus triple inhaler trial	Fluticasone furoate/ vilanterol 100/25 mcg, with or without umeclidinium (1218/2439)	ACQ≥1.5 despite maintenance therapy with medium-to-high-dose daily ICS plus LABA;; FEV1 ≥30–<85% of predicted and postBD FEV1 reversibility (≥12% and 200 ml); acute asthma symptoms requiring healthcare contact/change in therapy in last 12 months	Step 4: 1097 (100)	2.5 (0.6)	58 (13)	30 (18)	0.23 (0.91)†	20.0 (0.7)†
Benralizumab 2b trial (NCT01238861) [3] 52-week, randomized, controlled, double-blind, dose-ranging, Phase IIb clinical trial	Placebo +Maintenance therapy with moderate to high dose ICS and LABA (222/606)	medium-high dose ICS/LABA ≥1 year; prebronchodilator FEV1 ≥40% and <90% predicted; ACQ-6 score ≥1.5 on ≥2 occasions during screening; postbronchodilator FEV1 reversibility (≥12% and 200 ml) or a positive response to a methacholine challenge; 2-6 exacerbations in prior year	Step 4: 122 (55) Step 5 100 (45)	Eos High: 2.7 (1.0) Eos Low: 2.5 (0.8)	Eos High: 65 (15) Eos Low: 69 (15)	Eos High: 18 (15) Eos Low: 13 (13)	Eos High: 0.53 (30) Eos Low: 0.16 (0.09)	Eos High: 37.9 (31.9) Eos Low: 20.7 (13.9)

(supplementary table continues next page)

Couillard S, *et al. Thorax* 2022; 77:199–202. doi: 10.1136/thoraxjnl-2021-217325

… supplementary table

…Name	… Arm (n/N)	…Inclusion criteria	… GINA Step	…ACQ	…FEV1 % predicted	… PostBD change%	… Eos	… FeNO
PATHWAY (NCT02054130) [4] 52-week, randomized, double-blind, placebo-controlled, Phase II clinical trial	Placebo + Maintenance therapy with a moderate to high dose ICS and LABA (138/550)	ACQ-6 score ≥1.5 despite medium-to-high dose ICS and LABA for ≥6 months; prebronchodilator FEV1 ≥40% and <80% predicted; postbronchodilator FEV1 reversibility (≥12% and 200 ml); ≥2 exacerbations or ≥1 severe exacerbation resulting in hospitalization in the prior year;	Step 4: 73 (53) Step 5: 65 (47)	2.7 (0.7)	60 (14)	nd	0.38 (0.33)	37.8 (39.7)
STRATOS 1 (NCT02161757) [5] 52-week, randomized, double-blind, parallel-group, placebo-controlled, tralokinumab phase III clinical trial	Placebo +Maintenance therapy with moderate to high dose ICS and LABA (400/798)	Medium/high dose ICS+LABA ≥3 months; ≥2 exacerbations in prior year; prebronchodilator FEV1 <80% Predicted (<90% if aged 12-17); ACQ-6 score≥1.5; postbronchodilator FEV1 reversibility (≥12% and 200 ml)	Step 3: 3 (1) Step 4: 194 (49) Step 5: 203 (51)	2.6 (0·9)	62 (13)	23 (24)	0.25 (0.20)	29·6 (28·2)
STRATOS 2 (NCT02194699) [5] 52-week, randomized, double-blind, parallel group, placebo-controlled, tralokinumab phase III clinical trial	Placebo +Maintenance therapy with moderate to high dose ICS and LABA (422/837)	Medium/high dose ICS+LABA ≥3 months; ≥2 exacerbations in prior year; prebronchodilator FEV1 <80% Predicted (<90% if aged 12-17); ACQ-6 score≥1.5; postbronchodilator FEV1 reversibility (≥12% and 200 ml)	Step 3: 14 (3) Step 4: 196 (47) Step 5: 207 (50)	2.6 (0.9)	61 (15)	26 (25)	0.27 (0.23)	31.7 (27.2)

(supplementary table continues next page)

… supplementary table

…Name	… Arm (n/N)	…Inclusion criteria	… GINA Step	…ACQ	…FEV1 % predicted	… PostBD change%	… Eos	… FeNO
LIBERTY ASTHMA QUEST (NCT02414854)[6] 52-week randomised, double-blind, placebo-controlled, parallel-group trial	Placebo (1.14 mL and 2 mL) +Maintenance therapy with moderate to high dose ICS and ≤ 2 additional controllers (634/1902)	ACQ-5≥ 1.5 despite receiving medium-to-high-dose ICS plus up to two additional controllers; preBD FEV1 ≤80%; postBD FEV1 reversibility (≥12% and 200 ml); hospital presentation or treatment with systemic corticosteroids in last 12 months	Step 4: 293 (49) Step 5: 327 (51)	2.7 (0.7) and 2.8 (0.8)	58 (13) and 58 (14)	25 (19) and 26 (18)	0.37 (0.34) and 0.39 (0.42)	34.5 (28.5) and 38.4 (38.0)
DREAM (NCT01000506)[7] 52-week, multicentre, randomised, double-blind, placebo-controlled mepolizumab trial	Placebo +Maintenance therapy with high-dose ICS and LABA (155/616)	ACQ≥1.5 or prebronchodilator FEV1<80% predicted despite high-dose ICS and LABA for ≥12 months; postBD FEV1 reversibility (≥12% and 200 ml) or positive response to methacholine challenge; characteristic eosinophilic airway inflammation in previous year (≥1 of : sputum eosinophils >3%, peripheral blood eosinophils ≥0.3×10^9/L, FeNO>50ppb); ≥2 exacerbations in the prior year;	Step 5: 151 (100)	2.5 (1.1)	59 (15)	21 (nd)	0.28 (1.01)††	33.7 (0.8)††

Data are mean (SD) unless otherwise indicated; *median (range); † geometric mean (SD of log); †† geometric mean on $\log_e$ scale (SD). ACQ, asthma control questionnaire; Blood Eos, peripheral blood eosinophil count (×10^9 cells/L); FeNO, fractional exhaled nitric oxide (ppb); FEV1, forced expiratory volume in 1 second; ICS, inhaled corticosteroid; LABA, long-acting beta2-agonist; *n*, number of patients in the control arm; *N*, overall number of patients enrolled in trial; nd, not disclosed; BD, bronchodilator

Supplementary References

1 Beasley R, Holliday M, Reddel HK, *et al.* Controlled trial of budesonide-formoterol as needed for mild asthma. *N Engl J Med* 2019;**380**:2020–30. doi:10.1056/NEJMoa1901963

2 Lee LA, Bailes Z, Barnes N, *et al.* Efficacy and safety of once-daily single-inhaler triple therapy (FF/UMEC/VI) versus FF/VI in patients with inadequately controlled asthma (CAPTAIN): a double-blind, randomised, phase 3A trial. *Lancet Respir Med* 2021;**9**:69–84. doi:10.1016/S2213-2600(20)30389-1

3 Castro M, Wenzel SE, Bleecker ER, *et al.* Benralizumab, an anti-interleukin 5 receptor α monoclonal antibody, versus placebo for uncontrolled eosinophilic asthma: A phase 2b randomised dose-ranging study. *Lancet Respir Med* 2014;**2**:879–90. doi:10.1016/S2213-2600(14)70201-2

4 Corren J, Parnes JR, Wang L, *et al.* Tezepelumab in Adults with Uncontrolled Asthma. *N Engl J Med* 2017;**377**:936–46. doi:10.1056/NEJMoa1704064

5 Panettieri RA, Sjöbring U, Péterffy AM, *et al.* Tralokinumab for severe, uncontrolled asthma (STRATOS 1 and STRATOS 2): two randomised, double-blind, placebo-controlled, phase 3 clinical trials. *Lancet Respir Med* 2018;**6**:511–25. doi:10.1016/S2213-2600(18)30184-X

6 Castro M, Corren J, Pavord ID, *et al.* Dupilumab Efficacy and Safety in Moderate-to-Severe Uncontrolled Asthma. *N Engl J Med* 2018;**378**:2486–96. doi:10.1056/NEJMoa1804092

7 Pavord ID, Korn S, Howarth P, *et al.* Mepolizumab for severe eosinophilic asthma (DREAM): A multicentre, double-blind, placebo-controlled trial. *Lancet* 2012;**380**:651–9. doi:10.1016/S0140-6736(12)60988-X
